# Enhancing older adult financial decision making through the use of self-evaluation worksheets

**DOI:** 10.3389/fpsyg.2022.790088

**Published:** 2022-09-27

**Authors:** Natalie L. Denburg, Sam M. Collins, Norma P. Garcia, Prescott Cole

**Affiliations:** ^1^Department of Neurology, University of Iowa Carver College of Medicine, Iowa City, IA, United States; ^2^West Coast Office of Consumers Union, San Francisco, CA, United States; ^3^California Advocates for Nursing Home Reform, San Francisco, CA, United States

**Keywords:** financial, decision making, intervention, enhancement, aging, reverse mortgage

## Abstract

Financial products and options are frequently complex and difficult for consumers to understand, which, alongside positively oriented sales pitches and predatory practices, may lead to uninformed and hazardous financial decisions. While several legal reforms have been implemented to improve consumers’ understanding of financial products, these modifications have only achieved mixed results. An ongoing challenge is the passive nature of such modifications, giving rise to confirmation bias—noticing the information which confirms one’s belief about a product, while ignoring or not paying enough attention to the potential risks. The aim of this study was to test an implementable form of public policy to help older adults successfully navigate these financial decisions. We tested whether a self-evaluation worksheet (in conjunction with a written disclosure), involving an active manipulation of financial content, would significantly impact older adult participants’ responses in an experimental reverse mortgage scenario. Forty community-dwelling, healthy older adults were randomized to one of two conditions: Control Condition (passive manipulation of financial content) and Manipulation Condition (active manipulation of financial content). In addition to completing a comprehensive neuropsychological examination, participants were administered the Reverse Mortgage Task (RM Task). Results indicated that a simple yet active manipulation—imparting accurate and understandable information regarding a complex financial product that must be further manipulated—led to the following among participants in the Manipulation Condition: (1) declines in mood; (2) superior understanding of the financial product; and (3) reduced intention of purchasing the financial product. In order for older adults to be well informed about reverse mortgages, policies must be put in place to ensure that adequate and accurate information has been not only provided but also *actively processed* by the individual.

## Introduction

One’s ability to make sound financial decisions over the course of their lifetime is critical for economic, interpersonal, and psychological health and success. As with any large life decision, accurate comprehension is a key element in choosing the option that will best fit the individual. However, in the realm of financial decisions, the options and information available may be both foreign and difficult to understand. This, alongside positively oriented sales pitches, not to mention predatory practices, may lead to uninformed and detrimental financial decisions.

Several legal reforms have been implemented in an effort to increase the consumer’s understanding of complex financial products and transactions. Mandatory written disclosures are one such legal reform, implemented nationally in the case of home loans (3 FR 68241, Nov. 17, 2008, as amended at 76 FR 40616, July 11, 2011), and implemented in the state of California in the case of a reverse mortgage (California Legislative Assembly AB 329, 2009). However, we are now learning that written disclosures, while important, may be of limited usefulness in aiding comprehension and reducing the incidence of predatory lending practices, and these supporting data are both anecdotal and empirical (e.g., Stark et al., 2013).

Confirmation bias describes a phenomenon in which one gives more attention to information that confirms their already existing hypothesis than information that would contradict it (Costa et al., 2017). Even when disconfirming information is found, it is often undervalued compared to confirming information, suggesting that just the presentation of contradictory information on its own is not enough to prevent this bias (Oswald and Grosjean, 2004). This behavior can be observed in a variety of scenarios: social, scientific, political, and economic. Studies have investigated such behavior across a number of these settings, as it can often occur unintendedly and can dramatically influence the conclusion an individual reaches (Nickerson, 1998).

Confirmation bias is significant in large financial decisions, such as home loans and reverse mortgages, as individuals may be presented with highly confirmatory information by the provider before purchasing a financial product (Garcia et al., 2010). As an example, if an individual approaches a reverse mortgage believing that it will allow them to use the value of their home and continue to live in it, they may not pay enough attention to the potential risks associated with this product, only focusing on the information which confirms their beliefs. This can unintentionally or intentionally lead individuals to make decisions that are detrimental to their financial wellbeing. To prevent abuses, financial disclosure forms that present information more clearly for consumers, including potentially contradictory information, are sometimes provided in these situations. However, these financial disclosure forms may not be enough on their own to enable individuals to fully understand the conditions of a financial product or accurately judge its quality.

Changes to required home loan disclosure forms developed after the 2008 financial crisis increased overall recollection of loan terms and appropriately reduced participants’ ratings of loan quality for riskier loans. However, these changes did not significantly reduce individuals’ confirmation bias as shown by the attention (measured *via* eye-tracking) given to specific sections of the disclosure forms by participants (Stark et al., 2013). These forms attempted to present information more fully and clearly, but information that contradicted the initial loan terms was still potentially ignored. Additionally, the improvements to the home loan disclosure forms were largely lost when individuals were distracted during the process of reviewing these forms (Stark et al., 2013). Based on this, it is likely that disclosure forms are insufficient to adequately prepare one for these significant financial decisions, and a different strategy is needed to ensure a proper understanding of loan terms. In the current study, after the typical disclosure forms, participants in the Manipulation Condition were given a self-evaluation worksheet which required active manipulation of the information presented. With this additional requirement of active manipulation of the details provided about the financial product, it was expected that participants would gain a more in-depth understanding of the benefits and risks of the product presented, enabling them to make a better-informed decision.

A reverse mortage was the financial product of focus in our study. This financial option has grown in popularity in recent years and is frequently seen as an alternative to moving to an assisted living facility (Garcia et al., 2010). It is predicted that reverse mortgage products will continue to popularize as the baby boom generation shifts into the reverse mortgage market demographic. In principle, a reverse mortgage allows one to access the equity of their home without having to sell their home, requiring repayment of the mortgage after the death of the borrower, sale of the home, or foreclosure of the home. When originating a reverse mortgage, a portion of the home’s equity is consumed by start-up costs, any remaining mortgage debt, and insurance to protect the borrower if the house declines in value. These costs mean that borrowers cannot access the full equity of their home through a reverse mortgage, usually accessing 60–70% of the home’s equity after all fees (Nakajima, 2012).

There are potential benefits to reverse mortgages, specifically to individuals who are “house-rich, cash-poor” and do not want to sell their home, as it allows them to access the equity in their home while continuing to live in it. This has been viewed as a potential method to alleviate a lack of post-retirement income in aging societies (Knaack et al., 2020). Additionally, reverse mortgages are non-recourse loans, meaning that the amount of repayment due at the termination of the loan cannot exceed the value of the house. The non-recourse nature of reverse mortgages can protect the homeowner against decreases in the home’s value. If at the termination of the loan, the value of the house is less than the original evaluation for the reverse mortgage, the borrower/descendants are not obligated to pay for the difference, instead, this is typically covered by insurance (Nakajima, 2012).

However, there are significant potential risks to reverse mortgages that must also be considered. As an investment, home-ownership allows individuals to move money into a relatively illiquid form that cannot be accessed easily, therefore creating a “piggy bank” of savings. Reverse mortgages may effectively break this “piggy bank” as they allow homeowners to access the money they invested early. This may discourage appropriate savings from being set aside for retirement and is especially hazardous to relatively younger older adults who may outlive the equity they receive from a reverse mortgage (Nakajima, 2012). This hints at another risk of a reverse mortgage, that is if the borrower has to leave the home after a relatively short period of time, such as in the case of an unexpected medical incident requiring relocation to an assisted care facility. In such a case, the loan will be terminated and repayment will come due, and much of the equity the homeowner may have gained in selling their home may be lost in repaying the reverse mortgage. As such, in obtaining a reverse mortgage, homeowners are essentially “betting” that they will live in their home long enough to fully benefit from the loan, and as Nakajima (2012) states, some will lose this bet. This product can even have potentially disastrous outcomes: missing a home tax or insurance payment could lead to foreclosure and eviction and any non-borrower tenants of a home may be evicted after the death of the loanee (Brenner, 2017).

In a survey of over 1,700 households who were consulted for a reverse mortgage, Moulton et al. (2017) identified some of the primary self-reported factors for choosing whether or not to proceed with a reverse mortgage. For both those who did and did not get a reverse mortgage, “to gain extra income for everyday expenses” was the predominant motivation, along with “to pay off a mortgage,” which is consistent with the expectation that those considering a reverse mortgage would be “cash poor” and “house rich.” Those who did *not* originate a reverse mortgage were significantly more likely to list paying off their mortgage as the reason for their interest, nearly 20% of which stated they were behind on their mortgage payments at the time of counseling. Additionally, those who did not obtain a reverse mortgage were also more likely to report feeling under-informed about the product after counseling (Moulton et al., 2017).

Lack of financial literacy and a general misunderstanding of reverse mortgages are significant among consumers, as it is a relatively complicated financial product and is only accessible to older adults, who are more at risk to be experiencing age-related cognitive decline. Research has shown older adults to have the lowest level of financial literacy across all age groups. In a national survey of financial literacy, older adults were less likely to answer questions correctly and more likely to answer “don’t know” to a series of financial questions (Lusardi, 2012). This lack of financial literacy and product knowledge may also prevent individuals from sufficiently considering other financial options which are less costly than a reverse mortgage (Davidoff et al., 2017).

### Aim of study

The aim of this study was to test an implementable form of public policy. In particular, we tested whether a self-evaluation worksheet (in conjunction with a written disclosure), involving an active manipulation of financial content, would significantly impact older adult participants’ responses in an experimental reverse mortgage scenario.

## Materials and methods

### Participants

Forty participants were recruited from an existing research registry of 120 older adults in eastern Iowa. Participants in the current study were independently living, community-dwelling, and cognitively intact, with no history of neurological or psychiatric disease as determined secondary to extensive clinical interview (after Tranel et al., 1997). Participant age ranged from 65 to 88 years old (*Mean*_*age*_ = 77.13, *SD* = 6.31; *Median_*age*_* = 78 years) and the sample was 53% female.

### Procedure

Participants completed two independent visits. The first visit (Visit #1; 2 h in duration) consisted of completion of the comprehensive Neuropsychological Battery to confirm that participants were cognitively intact. The second visit (Visit #2; 45 min in duration) consisted of the RM Task. Twenty of the 40 older adults were randomized to the *Control Condition* (mean age = 78.7 years; 55% female) of the RM Task, while the remaining 20 older adults were randomized to the *Manipulation Condition* (mean age = 75.6 years; 50% female) of the RM Task. Informed consent was obtained for all testing procedures. Compensation was provided for participants at the rate of $15 per hour, for a total study payment of $41.25.

### Visit #1: Neuropsychological battery

#### Intelligence

Intellectual ability was measured using the Wechsler Abbreviated Scale of Intelligence—Second Edition (WASI-II; Wechsler and Hsiao-pin, 2011). Verbal intelligence (IQ) was assessed with the Vocabulary and Similarities subtests. In the Vocabulary subtest, participants are required to define words of increasing difficulty. In the Similarities subtest, participants are presented with two related words and asked to indicate how the two words are alike or similar. Performance IQ was assessed with the Block Design and Matrix Reasoning subtests. The Block Design subtest consists of several designs that the participant recreates using red and white colored blocks. The Matrix Reasoning subtest has the participant select one design from a five-alternative forced choice array to complete an incomplete pattern matrix. Full scale intelligence (IQ) was calculated from all four of the aforementioned subtests.

#### Language

Language abilities were measured using the short from of the Boston Naming Test (BNT; Kaplan et al., 2001), a 20-item test of confrontation naming abilities in which participants are shown line drawings, one at a time, and given 20 s to correctly name each object. The items consist of commonly encountered objects (e.g., bed) and less commonly encountered objects (e.g., artist’s palette).

#### Memory

Anterograde verbal memory was measured using the Rey Auditory-Verbal Learning Test (AVLT; Schmidt, 1996), which begins with five learning trials of a 15-word list. During each trial, the list is read aloud to the participant and he/she subsequently recites as many of the list words as possible. After a 30-min incidental delay, participants are again prompted to recite as many of the list words as possible, and this served as the memory variable of interest.

#### Attention and concentration

Attention and concentration were measured with both the Digit Span subtest from the Wechsler Adult Intelligence Scale—Fourth Edition (WAIS-IV; Wechsler, 2008) and the Benton Visual Retention Test (BVRT; Benton, 1946). The Digit Span test consists of two parts that are summed together into a total score. In the Digits Forward subtest, participants are read a number sequence and must recite the sequence directly as given. The Digits Backward subtest has participants recite the number sequence in reverse order. These subtests assess attention and concentration, respectively. The BVRT assesses visual attention and retention; 10 images containing one or more figures are individually presented for a 10-s time period, after which they are covered and immediately reproduced from memory by the participant. BVRT responses are scored with a number correct and a number of errors made, and the latter was utilized for data analysis.

#### Psychomotor speed

The Trail Making Test Part A (TMT-A; Reitan, 1958) assesses psychomotor speed. In the task, participants are instructed to draw lines connecting consecutively numbered circles on a sheet of paper as fast as they can without making errors. The examiner points out any errors made, and redirects the participant to the prior correct circle to resume. The score is the total number of seconds required to complete the task, with time taken for self-corrections as the penalty for committing errors.

#### Executive functioning

Executive functioning was assessed using the Trail Making Test Part B (TMT-B; Reitan, 1958), a set-shifting task, in which participants must alternate drawing lines between circles containing consecutive numbers and letters (e.g., 1, A, 2, B). Similar to TMT-A, participants are instructed to complete the task as quickly as they can without making errors. Errors are identified and corrected as in TMT-A. The score is the total time in seconds required to complete the task, again with time taken for self-corrections as the penalty for committing errors.

### Visit #2: Reverse mortgage task

**FIGURE 1:**
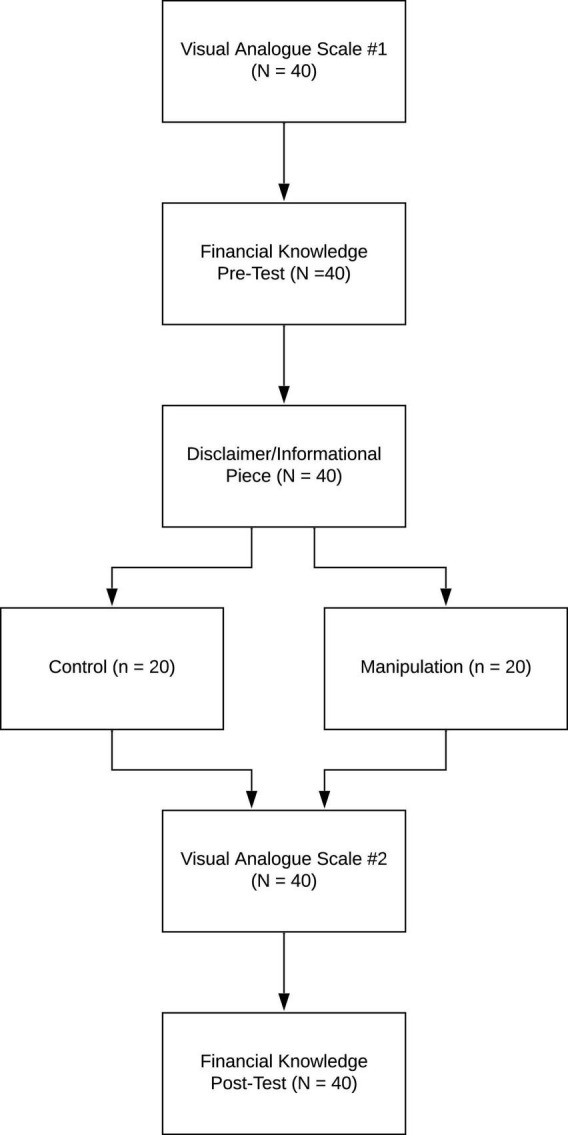
Outline of the procedures of the study.

The RM Task consisted of six segments. Regardless of randomized condition (Control vs. Manipulation), participants were provided the following disclaimer at the outset of Visit #2: “The following experiment is hypothetical, but to obtain the best information, we would like you to complete the following task as if you are interested in learning more about the stated financial product.” Of the six segments, all participants completed the initial three segments, involving a mood rating (Visual Analog Scale), completion of a financial knowledge quiz (Financial Knowledge Pre-Test), and reading of an informational piece on reverse mortgages (Informational Piece). Segment four contained the key experimental manipulation, in which participants were randomized into two conditions. The *Control Condition* consisted of participants who received only the required legal disclosure documents for the State of California as of 2008 (Important Notice and Written Checklist), which was followed by a 15-min, non-financial distractor task. The *Manipulation Condition* received both legal documents as well as a self-evaluation worksheet that required active manipulation of the content of a reverse mortgage (Reverse Mortgage Comprehensive Worksheet). All participants concluded the task by completing segments five and six, involving a second mood rating (Visual Analog Scale) and a financial knowledge post-test (Reverse Mortgage Comprehensive Assessment). The six segments are described in greater detail below and depicted in Figure 1.

1. Participants completed their first (of two) Visual Analog Scale to assess mood. On a visual scale from 0 to 100, with anchors at 0, 25, 50, 75, and 100, participants rated how they presently felt with regard to “happy,” “scared,” “calm,” and “irritated” (e.g., Irritated: 0, I don’t feel irritated at all; 25, I feel a little bit irritated; 50, I feel moderately irritated; 75, I feel quite a bit irritated; and 100, I feel extremely irritated). They indicated their level of positive emotionality (happy, calm) and negative emotionality (scared, irritated) by placing an X where they fell along the 0–100 continuum. Participants were encouraged to use the entire scale and not just the anchors.
2. Participants completed the Financial Knowledge Pre-Test. The pre-test consisted of 10 questions (six multiple-choice and four true-false questions), with five questions specific to reverse mortgage knowledge and five questions derived from the FINRA Financial Literacy Quiz.^1^
3. Participants were read a brief Informational Piece on reverse mortgages, taken directly from the California Civil Code Section 1923.5 on reverse mortgages (California Civil Code [CCC], 2011).
4. Participants were randomized into two conditions. The *Control Condition* consisted of participants who received only the current required legal disclosure documents for the State of California as of 2008 (Important Notice and Written Checklist), which was followed by a 15-min, non-financial distractor task. The *Manipulation Condition* received both legal documents, Important Notice and Written Checklist, followed by the self-evaluation worksheet that required active manipulation of the content of a reverse mortgage in order to respond to the worksheet’s questions (Reverse Mortgage Comprehensive Worksheet). The Worksheet contained 12 fill-in-the-blank sentences, along with a word bank. Although the 12 sentences were relatively straightforward, searching and re-reading the aforementioned Notice and Checklist, as well as consulting the word bank, were necessary to complete the task.
5. Participants completed their second (of two) Visual Analog Scale to assess mood.
6. Participants completed a final assessment, consisting of 22 questions, to test their knowledge and understanding of a reverse mortgage (Reverse Mortgage Comprehensive Assessment).

(The RM Task is available by request to the corresponding author).

### Statistical analyses

Preliminary analyses were conducted to examine the data for the presence of outliers and the appropriateness of assumptions of linearity, independence of errors, and multicollinearity. Independent samples *t*-tests were conducted to (1) examine the neuropsychological comparability of the two randomized groups, and (2) contrast the RM segments by group (i.e., Control vs. Manipulation Conditions). All statistical data analyses were performed using SPSS version 24.0 (IBM Corp., 2016).

## Results

Participants in the two conditions (Control vs. Manipulation) were identical in terms of age, sex distribution, years of completed education, and current-day cognitive abilities (e.g., intellect, memory ability, numeracy skills). These demographic and cognitive data are presented in Table 1, which indicate above average normal intelligence, as well as age-appropriate cognitive abilities per normative data (Lezak et al., 2012).

**TABLE 1:** Demographic and cognitive characteristics of the sample.

Category	Measure	Control mean (*SD*)	Manipulation mean (*SD*)	*p*-value
Demographics	*N*	20	20	–
	Age	78.7 (6.1)	75.6 (6.3)	*p* > 0.05
	Sex (% female)	55	50	*p* > 0.05
	Education	16.2 (3.1)	16.8 (2.7)	*p* > 0.05
Depression	BDI	4.6 (3.3)	4.0 (3.3)	*p* > 0.05
	Full scale IQ	118.4 (10.1)	119.1 (11.2)	*p* > 0.05
Mental status/intellect	Verbal IQ	116.7 (10.0)	118.1 (12.2)	*p* > 0.05
	Performance IQ	115.9 (14.2)	119.8 (11.5)	*p* > 0.05
Language	BNT	18.9 (1.3)	19.3 (0.6)	*p* > 0.05
Memory	AVLT delay	9.5 (2.5)	10.4 (3.5)	*p* > 0.05
Attention/concentration	Digit span total	17.4 (4.7)	16.8 (4.4)	*p* > 0.05
	BVRT errors	4.3 (2.8)	3.6 (1.6)	*p* > 0.05
Psychomotor speed	TMT A (time)	30.5 (8.4)	32.7 (8.6)	*p* > 0.05
Executive function	TMT B (time)	72.6 (23.6)	74.7 (33.8)	*p* > 0.05

BDI, Beck Depression Inventory; IQ, Intelligence Quotient; BNT, Boston Naming Test; AVLT, Rey Auditory-Verbal Learning Test; BVRT, Benton Visual Retention Test; TM, Trail Making Test.

Participants in the two conditions were also indistinguishable on the baseline study variables, as would be expected based on the randomization of participants to one of the two study conditions. More specifically, the following study variables, namely baseline emotional state (i.e., self-ratings on happy, scared, calm, and irritated), baseline financial knowledge, number of financial products purchased over the last 5 years, self-report of being content with the financial decisions they have made to date, and self-reported interest in financial products, were not reliably different when comparing the Control and Manipulation Condition samples (*p*s > 0.05).

By contrast, analysis of emotional state, reverse mortgage comprehension assessment, and rating of likelihood to purchase a reverse mortgage, all collected near the end of the RM task, revealed significant differences between the Manipulation and Control Conditions. More specifically, participants in the Control Condition and participants in the Manipulation Condition significantly differed on the following study variables, such that the Manipulation Condition (relative to Control Condition) displayed: lower feelings of “happy” during VAS #2 [*t*(38) = 3.14, *p* = 0.003]; lower feelings of “calm” during VAS #2 [*t*(38) = 8.31, *p* = 0.004]; higher feelings of “irritated” during VAS #2 [*t*(38) = –3.21, *p* = 0.003]; higher comprehension scores on the comprehensive assessment [*t*(38) = 4.47, *p* < 0.001]; and rated themselves as more unlikely to purchase a reverse mortgage [*t*(38) = –2.63, *p* = 0.012].

## Discussion

The present study examined how requiring participants to manipulate reverse mortgage disclosure information affected their comprehension, disposition, and emotional state related to the reverse mortgage. Currently, in everyday practice, general information about reverse mortgage options and risks are presented to individuals without evaluating their understanding of said information before making a financial decision. This leaves room for confirmation biases and miscomprehension of the disclosure forms, which may lead to potentially risky financial decision making.

In this study, we showed that a simple yet active manipulation – imparting accurate and understandable information regarding a complex financial product which, importantly, must be further acted upon—led to: (1) Changes in mood: relative to participants in the Control Condition, participants in the Manipulation Condition endorsed lower feelings of “happy,” lower feelings of “calm,” and higher feelings of “irritable,” after completing the self-evaluation worksheet; (2) Superior comprehension of the financial product: relative to participants in the Control Condition, participants in the Manipulation Condition displayed higher comprehension scores on the reverse mortgage comprehensive assessment administered at the conclusion of the study; and 3) Reduced intention of purchasing the financial product: relative to participants in the Control Condition, participants in the Manipulation Condition indicated that they were less likely to purchase a reverse mortgage in the future, when queried at the conclusion of the study.

Our findings, and the real-world repercussions, are made all the more important when one considers an age-related phenomenon known as the positivity effect. It refers to the tendency of older adults to focus on and demonstrate a bias toward positively valenced material (often together with low responsiveness toward negatively valenced material) (e.g., Carstensen et al., 1999). Empirical support for this bias has been established in multiple cognitive domains, and the data indicate that elders draw their attention away from (e.g., Mather and Carstensen, 2003) and show poorer memory for emotionally negative information (Denburg et al., 2003). Given an older adults’ diminished ability to attend to and remember negative content, modifications to the purchase of complex financial products, involving active manipulation of financial content, is imperative.

Our study is not without limitations. For one, the older adults included in our sample were high functioning, with more than 16 years of education, and intellectual functioning which fell in the high average range. It is possible that the high functioning nature of our sample could have biased our findings. Additionally, the sample size for this study was relatively small and results may not be representative of all older adults. Too, the financial purchase being made in this study was hypothetical, and as such participants’ opinions and decisions about the reverse mortgage product may have differed if they were considering a real (i.e., non-hypothetical) reverse mortgage. Participants were also making this financial decision in an environment free of distractions, whereas during an actual reverse mortgage purchase they may have the additional challenge of distractions (e.g., from a mortgage broker), as well as tangible economic pressures.

In summary, our data demonstrate that accurate understanding of a reverse mortgage impacts mood, comprehension, and purchase intention, all of which are crucial for sound financial decision making. Importantly, the lower purchase intention in the Manipulation Condition supports the notion that by requiring individuals to demonstrate understanding of reverse mortgage terms successfully, their likelihood of making a potentially financially risky decision decreases. As such, in order for older adults to be well informed about reverse mortgages, policy must be put in place to ensure that adequate and accurate information has been not only provided but also *actively processed* by the individual.

### Implications

This study provides evidence-based support to advance significant consumer protections in the law for a vulnerable population targeted for complex and costly financial products which are widely and aggressively marketed to all seniors, but appropriate for just a few. The study’s findings help inform policymakers of how important behavioral science is in determining what protections should be put in place for seniors considering reverse mortgages and why. As policymakers incorporate this knowledge into their decision making, they can craft policies, based on scientific evidence that provide critical information and an effective process for those considering reverse mortgages to determine whether the product is right for them, rather than banning the product all together. Ultimately, the study will help reinforce policies that encourage informed choice when it comes to reverse mortgages, and reduce the number of individuals and their families for whom a reverse mortgage is an inappropriate product that can lead to displacement and home loss for seniors and the loss of home equity for their families.

### Policy statement

The study described above led to the enactment of important legislation to combat the improper use of reverse mortgages among older adults, entitled Assembly Bill 1700 (AB 1700), filed with the California Secretary of State on September 30, 2014, and introduced by Assembly Member Medina, Chapter 854 (California Legislative Assembly AB 1700, 2014).

## Data availability statement

The datasets presented in this article are not readily available because of the anonymized data to protect the privacy of the individuals involved in the study. Requests to access the datasets should be directed to NG, natalie-denburg@uiowa.edu.

## Ethics statement

The studies involving human participants were reviewed and approved by the University of Iowa Institutional Review Board. The patients/participants provided their written informed consent to participate in this study.

## Author contributions

ND, NG, and PC contributed to conception and design of the study. ND and SC organized the database. ND performed the statistical analysis and wrote the first draft of the manuscript. ND, SC, NG, and PC wrote sections of the manuscript. All authors contributed to manuscript revision, read, and approved the submitted version.

## Footnotes

1
https://www.finra.org/financial_literacy_quiz
